# Robot-assisted radical cystectomy with intracorporeal reconstruction of urinary diversion by mechanical stapler: prospective evaluation of early and late complications

**DOI:** 10.3389/fsurg.2023.1157684

**Published:** 2023-06-13

**Authors:** Giovanni Cochetti, Alessio Paladini, Michele Del Zingaro, Sara Ciarletti, Francesca Pastore, Guido Massa, Lorenzo De Angelis, Ettore Mearini

**Affiliations:** Urology Clinic, Department of Medicine and Surgery, University of Perugia, Perugia, Italy

**Keywords:** robot-assisted radical cystectomy, intracorporeal urinary diversion, mechanical stapler, complications, perioperative outcomes

## Abstract

**Introduction:**

Radical cystectomy with pelvic lymph node dissection is the gold standard treatment for non-metastatic muscle-invasive bladder cancer and high-risk non–muscle-invasive bladder cancer. For years, the traditional open surgery approach was the only viable option. The widespread of robotic surgery led to its employment also in radical cystectomy to reduce complication rates and improve functional outcomes. Regardless of the type of approach, radical cystectomy is a procedure with high morbidity and not negligible mortality. Data available in the literature show how the use of staplers can offer valid functional outcomes, with an acceptable rate of complications shortening the operative time. The aim of our study was to describe the perioperative outcomes and complications associated with robot-assisted radical cystectomy (RARC) with intracorporeal urinary diversion (ICUD) using a mechanical stapler.

**Material and methods:**

From January 2015 to May 2021, we enrolled patients who underwent RARC with pelvic node dissection and stapled ICUD (ileal conduit or ileal Y-shaped neobladder according to the Perugia ileal neobladder) in our high-volume center. Demographic features, perioperative outcomes and early (≤30 days) and late (>90 days) post-operative complications according to the Clavien–Dindo classification, were recorded for each patient. We also analyzed the potential linear correlation between demographic, pre-operative as well as operative features and the risk of post-operative complications.

**Results:**

Overall, 112 patients who underwent RARC with ICUD were included with a minimum follow-up of 12 months. Intracorporeal Perugia ileal neobladder was performed in 74.1% of cases while ileal conduit was performed in 25.9%. The mean operative time, estimated intraoperative blood loss, and LOS were 289.1 ± 59.7 min, 390.6 ± 186.2 ml, and 17.5 ± 9.8 days, respectively. Early minor and major complications accounted for 26.7% and 10.8%, respectively. Overall late complications were 40.2%. The late most common complications were hydronephrosis (11.6%) and urinary tract infections (20.5%). Stone reservoir formation occurred in 2.7% of patients. Major complications occurred in 5.4%. In the sub-analysis, the mean operative time and the estimated blood loss improved significantly from the first 56 procedures to the last ones.

**Conclusion:**

RARC with ICUD performed by mechanical stapler is a safe and effective technique. Stapled Y-shaped neobladder did not increase the complication rate.

## 1. Introduction

Radical cystectomy (RC) with pelvic lymph node dissection is the gold standard treatment for patients with non-metastatic muscle-invasive bladder cancer and selected patients with high-risk non–muscle-invasive bladder cancer, providing the best oncological results (1, 2). The quality of life (QoL) is often altered in these patients due to the severe disease, the high-complexity surgery they undergo, the complications of which RC is burdened, the type of urinary diversion performed, and the aesthetic results.

Usually, the surgical strategy and the types of urinary diversions are determined according to several factors, such as comorbidities, gastrointestinal system status, the site of the bladder cancer and the pre-operative renal function. Performance status and life expectancy shall also influence this choice (3).

For years, the traditional open surgery approach was the only viable option. It is usually related to severe blood loss, long length of stay, slow recovery, high rate of complications, and poor aesthetic results. The widespread of robotic surgery led to its employment also in RC in order to reduce complication rates and improve functional outcomes (4, 5).

In particular, there are several studies, in the literature, supporting robot-assisted radical cystectomy (RARC), which has shown comparable oncological and functional outcomes to open radical cystectomy (ORC) (6, 7).

However, morbidity remains high in both open and robotic approaches. Robotic, in fact, is burdened by longer operative time, complications due to steep Trendelenburg and high CO_2_ pressure. In order to reduce the risk of these factors, the use of a mechanical stapler, for intracorporeal urinary diversion, has been proposed.

The reconstructive time of a urinary diversion surgery is certainly a delicate and challenging time of the operation. Data available in the literature show how the use of staplers can offer valid functional outcomes, with an acceptable rate of complications shortening the operative time (8). The aim of our study was to describe the complications associated with RARC with intracorporeal urinary diversion (ICUD) using a mechanical stapler. We analyzed the first 56 patients in comparison to the last 56 to show the differences encountered during the learning curve of this high-complexity surgery.

## 2. Materials and methods

From January 2015 to May 2021, 146 patients underwent RARC with pelvic node dissection in our high-volume center for non-metastatic muscle-invasive or high-risk non–muscle-invasive bladder cancer. Neoadjuvant, platinum-based chemotherapy was selectively adopted according to treating physicians' and patients’ characteristics and preferences. An ICUD was performed in 126 patients: non-continent ileal conduit according to the Bricker technique or continent ileal Y-shaped neobladder according to the Perugia ileal neobladder (PIN) yet described with an open approach (9).

All the procedures were performed by a single experienced robotic surgeon (EM) using the daVinci Si or Xi surgical system. The case volume of the surgeon was between 25 and 30 RARC per year, and in his surgical experience, he performed over 1,500 robotic procedures.

### 2.1. Surgical procedure and technical aspects

The patient was placed in a dorsal lithotomy position at 25° of Trendelenburg using the Pink Pad® (Xodus Medical Inc.). Four robotic trocars were placed: two in line with the supraumbilical optic trocar, 8 cm from the optical one, and another 8 cm from the right one, on a transverse line 2 cm cranial to the umbilicus. A 12 mm AirSeal® (CONMED Corporation) trocar was placed on the left patient side, in line with the most lateral right trocar, for the bed assistant surgeon. A 12 mmHg intraperitoneal insufflation pressure was maintained during the procedure, thanks to the AirSeal system.

RARC was performed in all cases, and nerve-sparing and vaginal-sparing surgery in males and females, respectively, was carried out only in selected cases. To control vessel bleeding, Hem-o-lok® clips and bipolar forceps were used. Ureters were dissected and transected next to the bladder previous to their closure with clips. In all patients, an extended pelvic lymph node dissection was performed using clips and bipolar energy to seal lymphatic vessels.

### 2.2. Bricker ileal conduit

It was performed using approximately 15 cm of ileum, starting at 20 cm from the ileocecal valve. Ileal resection was performed with the 60 mm mechanical ECHELON FLEX™ ENDOPATH™ stapler. Bowel continuity was restored in a latero-lateral manner using the stapler and intestinal mesentery was sutured. The left ureter was passed under the mesosigma. Ureters were spatulated on their anterior side, and 7 Ch double J stents were placed. On the antimesenteric side of the ileal segment, a direct uretero-ileal anastomosis was performed with 4-0 absorbable barbed suture that started at the angle of the ureteric spatulation and ran on both sides. The distal extremity of the ileal conduit was secured to the skin with interrupted long-lasting absorbable suture.

### 2.3. Y-shaped Perugia ileal neobladder

It was performed using approximately 45 cm of ileum, starting at 20 cm from the ileocecal valve. Ileal resection was performed with the 60 mm mechanical ECHELON FLEX™ ENDOPATH™ stapler after pulling the ileal segment toward the urethral stump to assure a urethral-ileal anastomosis tension-free. Bowel continuity was restored in a latero-lateral manner using the stapler and intestinal mesentery was sutured. The ileal segment was folded on itself in a “Y” shape. An incision of 1 cm was performed in the lowest part of the ileal segment which was secured to the urethral stump. Two incisions, each of about 1 cm, were performed in the distal antimesenteric portion of the “Y” arms to insert the mechanical stapler to detubularize downward the ileum. The urethral-ileal anastomosis was carried with a 2/0 long-lasting absorbable barbed running suture. The left ureter was passed over the mesosigma. Ureters were spatulated on their anterior side, and 7 Ch double J stents were placed. On the antimesenteric side of the “Y” arms, a direct uretero ileal anastomosis was performed with 4-0 absorbable barbed suture that started at the angle of the ureteric spatulation and ran on both sides. A 20 Ch Foley catheter was left in place for 14 days in the neobladder.

In both urinary diversions, an intraperitoneal drain and a pelvic one were left in place for 7 and 14 days, respectively. All patients were post-operatively managed according to the enhanced recovery after surgery (ERAS) protocols.

### 2.4. Data collection

Data were prospectively collected after obtaining written informed consent from every participant before surgery.

Age, gender, American Society of Anesthesiology (ASA) score, and Eastern Cooperative Oncology Group (ECOG) were used to classify performance status and patients' functional status, pre-operative grade of hydronephrosis and renal function impairment, operative time (OT), estimated blood loss (EBL), length of hospital stay (LOS), type of urinary diversion, rate of conversion, and early (≤30 days) and late (>90 days) post-operative complications according to the Clavien–Dindo classification, were recorded for each patient.

Patients were evaluated at 1, 3, and 6 months after the surgery, then every 6 months until the third year, and annually thereafter with CT scan images, blood exams, arterial blood gas analysis, and urinary cytology. Those with a higher risk of urethral involvement were followed up with urethroscopy at 3 and 6 months post-surgery, then every 6 months until the third year, and annually thereafter.

Patients with previous pelvic surgery for cancer and abdominal and pelvic radiation therapy and those without a minimum follow-up of 12 months were excluded from the study.

We analyzed the potential linear correlation between demographic, pre-operative and operative features and the risk of complications. We reported functional outcomes such as urinary continence and sexual potency. Urinary daytime and nocturnal continence were evaluated, at 30 post-operative days, in patients with orthotopic neobladder (ONB) through direct interview according to question number 3 of the Expanded Prostate Cancer Index Composite (EPIC) questionnaire (10). Pre-operative sexual function was assessed in all males using the five-item International Index of Erectile Function (IIEF-5) (11). Only in pre-operative potent patients, defined as IIEF-5 score was ≥17, who received PIN, we reassessed erectile function at 30 post-operative days with IIEF-5 score.

Two sub-analyses were performed comparing perioperative and complications outcomes of the first 56 procedures (Group 1) with the second 56 procedures (Group 2) and to compare neobladder and ileal conduit.

The Internal Review Board of the University of Perugia approved the study protocols, and all subjects signed an informed consent. The investigation conformed to the principles outlined in the Declaration of Helsinki (1997).

### 2.5. Statistical analysis

Mean with standard deviation (SD) and median with interquartile ranges (IRQ) were used to report continuous variables and frequencies for categorical variables. The Pearson correlation coefficient was used to measure the strength of a linear association between variables, and statistical significance was set at *p* < 0.05. For the analysis of discrete variables, values were reported as a percentage of total subjects in each study group, and the contingency test (chi-square test) was used to calculate statistical significance. For the analysis of continuous variables, the Kolmogorov–Smirnov (KS) normality test was performed to analyze the distribution of data. *p*-values were calculated using the Student’s *t*-test (unpaired *t*-test with Welch's correction) for normally distributed data (KS test passed) and the Mann–Whitney test for data with skewed distribution (KS test failed). The statistical significance was set at *p* < 0.05. Statistical analysis was conducted using SPSS® Statistics Software.

## 3. Results

Overall, 126 patients underwent RARC with ICUD, and we included 112 patients, of which 98 were males (M) and 14 were females (F), without previous pelvic surgery for cancer or abdominal and pelvic radiation therapy, with a minimum follow-up of 12 months.

The mean age of the population was 66.2 ± 7.9 years. A total of 81 (72.3%) were between 50 and 70 years old, and 31 (27.7%) were older than 70 years. The median ASA score was 3 ± 1. Patients with preoperative hydronephrosis were 20 (17.9%); four suffered from mild renal impairment and two from moderate one (Table 1). A total of 25 male patients were pre-operatively potent.

**Table 1:** Pre-operative characteristics.

	Mean ± SD/median ± IRQ/%
Age (years)	66.2 ± 7.9
Male	66.1 ± 7.3
Female	66.2 ± 8.2
BMI (kg/m^2^)	
<18.5	4.4%
18.5–24.9	33.9%
25.0–29.9	58.0%
>30	3.7%
ASA score	3 ± 1
ECOG	0 ± 1
Hydronephrosis	17.9%
Renal impairment	
Mild	3.6%
Moderate	1.8%
Severe	0%

The mean OT was 289.1 ± 59.7 min. The EBL was 390.6 ± 186.2 ml. Table 2 shows the intraoperative outcomes. The overall rate of transfusion was 13.4%. In five (4.5%) males and two (1.8%) females, a nerve-sparing procedure or vaginal- and nerve-sparing surgery were performed, respectively. The mean LOS was 17.5 ± 9.8 days.

**Table 2:** Intraoperative outcomes.

	Overall (*n*. 112)	PIN (*n*. 83)	Ileal conduit (*n*. 29)
Operative time (min)	289.1 ± 59.7	306.6 ± 67.3	267.2 ± 50.7
EBL (ml)	390.6 ± 186.2	406.1 ± 103.5	380.3 ± 241.3
Intraoperative complications	0%	0%	0%
Rate of conversion	0%	0%	0%

All patients were treated with the ERAS protocol principles. Time to bowel was 4.3 ± 2.7 days, time to diet was 6.4 ± 3.1 days, and time to first mobilization was 1.8 ± 0.7 days.

Intracorporeal PIN was performed in 74.1% of cases while the non-continent ileal conduit was performed in 25.9%. On average, ureteral stents were left in place for 22.4 ± 6.4 days.

For PIN, the mean OT was 306.6 ± 67.3 min, and the EBL was 406.1 ± 103.5 ml. The ureteral stents were left in place on average for 22.6 ± 12.4 days. Daytime continence was reached in 74.7% of patients, while nocturnal continence in 36.1%. Potency was maintained in five male patients out of 25 pre-operatively potent (20%), with the use of PDE5-I drugs.

For the ileal conduit, the mean OT was 267.2 ± 50.7 min, and the EBL was 380.3 ± 241.3 ml. The ureteral stents were left in place on average for 20.7 ± 11.3 days.

Overall, in the first 30 post-operative days, 62.5% of patients had no complications. Minor complications (grade I–II) accounted for 26.7% of cases, and major complications (grade III–V) accounted for 10.8%. The most frequent early complication encountered was urinary tract infection, which affected 22 patients (19.6%) treated with antibiotics. Early major complications recorded were hydronephrosis in 4.5% of patients for stent displacement and acute kidney injury with associated urinary tract infections in 6.3% due to stent occlusions. In all cases of major complications, we performed anterograde stent replacement.

Overall late complications (>90 days) were 40.2%. The late most common complications were hydronephrosis (11.6%) and urinary tract infections (20.5%). Major complications occurred in six patients (5.4%) showing hydronephrosis and urosepsis: four underwent anterograde stent placement (grade IIIa), one patient underwent ureteral reimplantation (grade IIIb), and one patient died of septic shock (grade V).

Cancer recurrence was recorded in four patients, in one patient at 3 months post-surgery, and in three patients at 6 months. Three patients died from BCa during the follow-up.

According to the statistical analysis, no demographic, pre-operative, and operative data correlated with the risk of complications (Table 3).

**Table 3:** Correlation between pre-operative and operative data with complications.

	Early complications (*p*-values)	Late complications (*p*-values)
Age	0.48	0.48
Hydronephrosis	0.36	0.56
ASA	0.22	0.39
Operative time	0.43	0.78

In the sub-analysis, the mean OT dropped from 352.7 ± 70.8 min for the first 56 procedures to 268.8 ± 54.3 min in Group 2, with significance (*p* = 0.04). The EBL changed significantly from 578.4 ± 186.1 ml to 332.5 ± 172.6 ml (*p* = 0.03), respectively, for Group 1 and Group 2. In the 56 RARC, the transfusion rate was 17.9%, and it dropped to 8.9% in the Group 2, without significance. Concerning LOS, no significant difference was found between the two groups, even if it was slightly lower in Group 2 with respect to Group 1 (15 vs. 17,5, *p* > 0.05). The number of overall complications (48 vs. 39) and major complications (12 vs. 6) was higher in Group 1 but without significance Table 4.

**Table 4:** Comparison between the first 56 (Group 1) and the last 56 (Group 2) procedures.

	Group 1	Group 2	*p*-value
Operative time (min)	352.7 ± 70.8	268.8 ± 54.3	***0*** *.* ***04***
EBL (ml)	578.4 ± 186.1	332.5 ± 172.6	***0***.***03***
*n*. overall complications	48	39	0.39
*n*. grades III–V	12	6	0.19

In bold significant values.

In the sub-analysis performed between neobladder and ileal conduit, in the early complications, minor ones occurred in 27.7% of the neobladder group and 24.1% of the ileal conduit group (*p* = 0.71), while major in 10.8% vs. 10.3% (*p* = 0.94). For late complications, minors occurred in 43.4% of the neobladder group and 31% of the ileal conduit group (*p* = 0.45), while major ones in 6% vs. 3.4% (*p* = 0.60). Between these two groups, there was no significance in the complications analysis.

## 4. Discussion

ORC with bilateral lymph node dissection is the standard treatment for patients with non-metastatic muscle-invasive bladder cancer and recurrent high-risk non–muscle-invasive bladder cancer with the objective of radicality of the treatment (12–14).

However, the robotic approach is a valuable alternative. In the available RCT of comparison between ORC and RARC, the latter proved to be advantageous in terms of lower transfusion rate and shortened hospital stay; moreover, comparable perioperative and oncological outcomes, complication rate, and QoL were comparable between the two techniques, while RARC showed a longer operative time. Unfortunately, data of high-level evidence on RARC with ICUD are poor, in particular, those on intracorporeal neobladder urinary diversion (15). In our series, we performed 83 orthotopic ileal Y-shaped neobladder and 29 ileal reservoir procedures using a mechanical stapler.

The main key point and the originality of our work consist of the prospective evaluation of perioperative outcomes and complications rate of RARC with ICUD using a mechanical stapler. The safe use of a stapler for ileo-ileal anastomosis has been yet debated in the literature, and it is routinely performed. Indeed, the stapler anastomosis ensures waterproofing sutures allowing to reduce the risk of leakage and dehiscence; with respect to the handsewn technique, the stapler anastomosis has either the same or a lower percentage of leakage (16, 17). Whereas the fecal material in the small bowel is almost liquid, we feel that stapled urinary diversion reconstructions are at least as safe as those performed by hand. In our opinion, the use of a stapler for the reconstruction of ICUD may simplify the technique ensuring leakproof closure and reducing the OT as well as all complications related to prolonged pneumoperitoneum in a steep Trendelenburg position. Indeed, a steep Trendelenburg increases the risk of cardiopulmonary complications, in addition to those related to the body position (18, 19).

Recently, Barbalat et al. (20) reported a saving of about 1 h of OT and almost $ 3,000 per patient, thanks to the use of a stapler for reservoir reconstruction. The OT for ORC with reconstruction urinary diversion is generally reported as shorter (2.5–5.6 h) using a mechanical stapler than for handsewn (4.3–7.9 h) reservoirs (9, 21–23). Concerning the robotic approach, the mean OT for intracorporeal ileal conduit ranged from 240 to 510 min with respect to extracorporeal one ranging from 270 to 470 min. Otherwise, the mean OT for RARC with intracorporeal neobladder reconstruction was between 305 and 477 min compared to 444–669 min for extracorporeal one (24). Our findings revealed a mean OT of 289.1 ± 59.7 min, which is comparable to other experiences with open stapled reservoirs (25).

However, analyzing the mean OT between the first 56 RARC and the last ones, we reported a significant reduction (353 vs. 269 min, *p* < 0.05). In their recent prospective study, Checcucci et al. (26) reported the results from 45 RARC with an intracorporeal reconstruction of a Y-shaped handsewn neobladder. The median OT was 287 min (range 263–315 min), of which 165 min (151–182 min) was the reconstruction time.

Concerning the other perioperative outcomes, our findings revealed a mean EBL of 391 ml, which was in line with the results reported in the literature. In a review concerning the outcomes of RARC with ICUD, Martin and Corcoran (24) reported an EBL of 200–400 ml. Similarly, the Pasadena Consensus Panel reported an EBL ranging from 200 to 430 ml with a 10%–53% transfusion rate for RARC with ICUD (27). However, in our series, the mean EBL was lower in the last 56 RARC than that in the first 56 (578 vs. 333 ml, *p* < 0.05).

The Pasadena Consensus Panel suggested an LOS of 5–10 days for very experienced surgeons (>100 cases) (27). Our findings showed an LOS of 17.5 days which is higher than other series. This finding could be due to different factors: (1) Data on LOS after RC are often difficult to interpret because LOS is affected by different institutional policies and healthcare environments of the country, specially related to the absence of local health support structures. Indeed, in an Italian series of RARC with intracorporeal neobladder, the authors reported an LOS of 15 days that is similar to our results (26). (2) We prefer to discharge the patient with orthotopic neobladder only after catheter removal in order to guarantee frequent irrigation of urinary catheters to clear the neobladder of mucus and after educating him about new urination methods, while in a patient with ileal reservoir, after instructing about the management of urostomies. We feel that this strategy could facilitate the post-operative course, reduce early readmission rate, and accelerate the return to normal daily activities, reducing the psychological impact of such a destructive intervention. (3) LOS could be partly influenced by surgical experience. In our sub-analysis, LOS showed a decreasing trend with increasing surgical experience, even if no significant difference was found between the two groups. Similarly, in a randomized controlled trial comparing 58 ORC with 58 RARC with ICUD, the authors reported a median LOS of 7 days (range 6–9), whereas in the interim analysis of the first 58 consecutive patients undergoing RC (30 RARC vs. 28 ORC) included in the same randomized controlled trial, the authors reported mean hospital stay of 10 days (±11) (28, 29).

Functional outcomes of our series appeared to be in line with those of the literature, in particular for urinary continence (30). The low rate of potent males could be addressed to the patients' mean age, the small number of pre-operatively potent patients and the low rate of nerve-sparing surgery.

Regardless of the type of surgical approach, RC is a very complex surgical procedure characterized by not negligible mortality and high morbidity (31, 32). Three long-term studies and one population-based cohort study reported mortality within 30 days and 90 days of 1.2%–3.2% and 2.3%–8.0%, respectively (33–35). In our series, the perioperative and post-operative mortality was very low: no death was registered at 30 days, while at 6 months, it was only one (0.93%).

Contemporary series reported post-operative complication rates for RARC with any type of urinary diversion ranging from 34% to 52% (36–39).

In a multi-institutional database of 939 patients who underwent RARC with urinary diversion performed *via* an extra- or intracorporeal approach, the authors reported a total complication rate of 41% at 30 days and 48% at 90 days. Specifically, among 90-day complications, 29% were of low grade (1–2 according to Clavien–Dindo classification), and 19% were of high grade (3–5). Reoperation within 30 days from surgery was necessary for 53/939 patients (5.6%) due to fascial dehiscence, small bowel obstruction, urine leakage, and bleeding (40).

Recently, Simone et al. (41) described 180-day complications in 45 patients undergoing RARC with intracorporeal handsewn ileal neobladder. The findings showed 35% of high-grade complications including two (4.4%) large and one (2.2%) symptomatic lymphoceles requiring surgical treatment and percutaneous drainage, respectively, five (11.1%) hydronephrosis requiring mono/bilateral nephrostomy, three (6.7%) bowel occlusions surgically treated, and one case of anastomosis stenosis that needed of ureteral reimplantation.

Our findings showed an overall complication rate of 77.7%. Specifically, complications at 30 days were 37.5% of which 10.8% were high-grade. Late complications were 40.2%, and those of high grade were 5.4%. This result is in line with those reported in recent major series. The most common complication was urinary tract infections, including those who needed hospital readmission and intravenous antibiotic therapy: they occurred in 25.9% within 30 days and 20.5% later than 30 days, which are comparable to data in Literature.

The peculiarity of our technique is the use of a mechanical stapler to build ICUD. However, the role of a stapler in performing the urinary diversion has long been debated in literature regarding the risk of stone formation, and it has not yet been clarified. Recently, Muto et al. (8) reported perioperative and functional outcomes of 606 patients undergoing ORC with stapled ileal neobladder according to the Camey II technique. Indeed, neobladder was carried out using 45 cm of ileum and configured with Y-shape. In their series, the authors revealed an overall complication rate of 51.2%, of which 24.3% occurred within 90 days from surgery and 26.9% later than 90 days. Specifically, the most common late diversion-related complication was neobladder stones which occurred in 4.5% of patients after a median of 36 months. In our series, stones of reservoirs occurred in three patients (2.7%), all with intracorporeal neobladder, and were easily removed in an outpatient setting. Our stone rate is lower than those reported in literature, and this could be due to two factors: (1) the Y-shaped neobladder was performed using 40–50 cm of ileum resulting in a smaller and elongated reservoir with a lower initial volume. This feature may result in an initial higher rate of urinary incontinence, but it could reduce the risk of urinary retention and, consequently, of stone formation. Indeed, unlike the other technique of reservoir configuration, stapled Y-shape neobladder is less prone to progressive enlargement and emptying failure. (2) Our median follow-up is slightly shorter than those of other series. Indeed, the incidence of neobladder stones reported in literature is commonly higher in stapled (6%–16%) than in handsewn (0%–8%) reservoirs, and it increases with time: longer follow-up is associated with higher stone formation (8). Ferriero et al. (23) described their retrospective experience reporting a stones rate of 8.1% in 445 Padua stapled ONB, during a median follow-up of 41 months, even though this rate increased up to 25% after 15 years according to the Kaplan–Meier method.

One of the most common and critical complications after RARC with ICUD is benign ureteroenteric anastomotic strictures (UES). UES could be the result of iatrogenic ischemia caused by excessive handling with robotic instruments of the ureter, causing damage to the adventitia, tension, and trauma during the dissection and reimplantation times. The strictures are asymptomatic in 75% of cases but could lead to hydronephrosis, colic pain, urine infection, and renal impairment. In the literature, the incidence of UES varies according to study design, surgical technique, and experience (42). Narita et al. in their review reported a rate of incidence ranging from 3% to 25.3%, in the study focused on UES, but at the beginning of the RARC learning curve, that incidence could reach pikes of 47% (43, 44). No significative differences were reported by Hosseini et al. in the case of ileal conduit or neobladder and from the International Robotic Cystectomy Consortium comparing ECUD and ICUD (45, 46).

In our series, where we have considered also the cases treated during the learning curve, UES were recorded in 10.8% of cases as early complications and in 11.6% as late complications, in line with data from the literature. The complication rate in a such challenging procedure should be related to the surgeon's experience and learning curve (47). From our database, we can state that RARC with ICUD represents a challenging procedure also in expert hands, and a learning curve is demanded. In fact, only after about 50 procedures OT and EBL were significantly reduced while complications were lower but without statistical significance. These data confirmed the learning curve length of about 50 procedures as yet reported in the literature (48). In our series, the high robotic experience of the surgeon may have impacted the no significant differences in the first 56 procedures vs. the last 56 procedures.

The main strength of our work is that perioperative outcomes and complications rate of RARC with ICUD performed using a mechanical stapler were prospectively collected and analyzed. To our knowledge, no prospective study analyzing the perioperative outcomes of RARC with an intracorporeal reconstruction of urinary diversion by stapler was reported in the literature.

However, we have to consider some main limitations of our study such as the small sample size, the short follow-up, and the absence of a comparison handsewn ICUD group.

## 5. Conclusion

RARC with ICUD performed by a mechanical stapler is a safe and effective technique. Stapled urinary diversions are not associated with a higher risk of stone reservoir formation or other complications.

## Data Availability

The raw data supporting the conclusions of this article will be made available by the authors, without undue reservation.
